# Argonaute2 attenuates active transcription by limiting RNA Polymerase II elongation in *Drosophila melanogaster*

**DOI:** 10.1038/s41598-018-34115-1

**Published:** 2018-10-24

**Authors:** Ezequiel Nazer, Ryan K. Dale, Cameron Palmer, Elissa P. Lei

**Affiliations:** 1 Nuclear Organization and Gene Expression Section, Bethesda, USA; 2 Laboratory of Cellular and Developmental Biology, National Institute of Diabetes and Digestive and Kidney Diseases, National Institutes of Health, 9000 Rockville Pike, Bethesda, MD 20892 USA

## Abstract

Increasing lines of evidence support that Argonaute2 (AGO2) harbors several nuclear functions in metazoa. In particular, *Drosophila* AGO2 modulates transcription of developmentally regulated genes; however, the molecular mechanisms behind AGO2 recruitment into chromatin and its function in transcription have not been deeply explored. In this study, we show that *Drosophila* AGO2 chromatin association depends on active transcription. In order to gain insight into how AGO2 controls transcription, we performed differential ChIP-seq analysis for RNA Polymerase II (Pol II) upon depletion of AGO2. Remarkably, we find specific accumulation of the elongating but not initiating form of Pol II after AGO2 knockdown, suggesting that AGO2 impairs transcription elongation. Finally, AGO2 also affects Negative Elongation Factor (NELF) chromatin association but not the Cyclin Dependent Kinase 9 (CDK9). Altogether, these results provide key insights into the molecular role of AGO2 in attenuating elongation of certain actively transcribed genes.

## Introduction

*Drosophila* AGO2 has been studied as paradigm for Argonaute proteins as an effector of the short interfering (siRNA) and microRNA pathways. In addition, *Drosophila* AGO2 has been used as metazoan model to explore its nuclear non-canonical functions such as transcription, splicing and DNA repair^1–4^, which are also conserved in humans^5^. Previous ChIP-seq analysis showed that AGO2 is mainly associated with promoters, enhancers and insulators^6^. Furthermore, AGO2 is known to be required for proper chromatin looping between the enhancer and promoter of the homeotic gene *Abd-B*, thus affecting its transcription^6^. Importantly, the function of AGO2 in transcription and chromatin topology has been shown to occur independently of the siRNA pathway^3,6,7^.

Genome-wide transcriptional analyses by nascent euRNA-seq (neuRNA-seq) and steady-state RNA-seq revealed that depletion of AGO2 primarily causes up-regulation of certain active promoters^3^. Moreover, it has been shown that LaminB, a key component of the nuclear lamina, also modulates AGO2-dependent genes likely by controlling chromatin topology^3^. Comparisons with several chromatin features determined that such affected promoters are bound by AGO2 and associated with RED chromatin. This chromatin class has been characterized as a hallmark for active transcription yet is depleted of H3K36me3, a histone modification associated with transcription elongation^8^. In addition, RED chromatin is associated with genes that are susceptible to modulation during development as well as external signals^8^. In this vein, AGO2 was found to interact physically with the Pol II complex^1,3^ and the Negative Elongation Factor (NELF)^1^, a factor implicated in transcriptional pausing^9,10^. Although NELF has been characterized to inhibit transcription elongation *in vitro*^1,9,10^, it can also play a positive role in transcription by keeping promoters in a primed, open state to allow for regulated activation^11^. It remains an open question as to whether AGO2 and NELF interact functionally.

In this study, we focused our efforts on elucidating the molecular mechanism driving AGO2 chromatin association as well as how AGO2 attenuates transcription. Our ChIP-seq analysis showed that AGO2 recruitment into chromatin depends on active transcription. Strikingly, we found that AGO2 impairs the ability of Pol II to elongate and alters NELF-E recruitment at active promoters. Overall, these results provide molecular insights into the mechanisms behind AGO2 recruitment into chromatin and the role of AGO2 in fine-tuning active transcription.

## Results

### AGO2 recruitment into chromatin depends on active transcription at a proportion of AGO2-bound promoters

**Figure 1:**
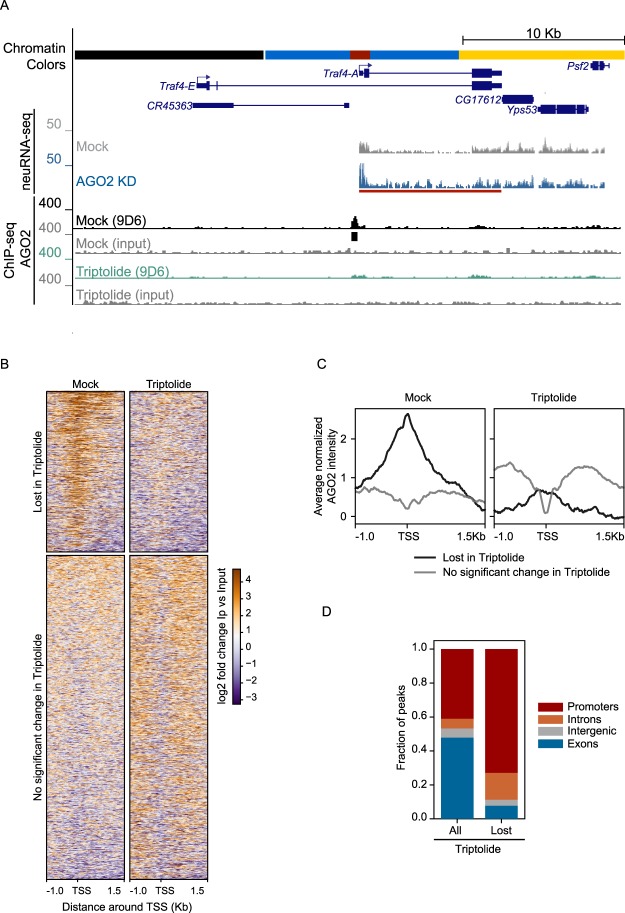
AGO2 recruitment into chromatin depends on active transcription at a proportion of AGO2-bound promoters. (**A**) Screenshot showing an example AGO2-dependent gene *Traf4-A*, where AGO2 ChIP signal is lost upon addition of the transcription inhibitor Triptolide. Black bar corresponds to ChIP-seq peak. Red bar corresponds to significant neuRNA-seq transcriptional up-regulation upon AGO2 KD. Note that the active TSS is located within RED chromatin. (**B**) Heat map of AGO2 lost or unchanged peaks upon Triptolide treatment sorted by decreasing average ChIP-seq signal. (**C**) Metagene analysis for average normalized AGO2 ChIP-seq signal for sites in (**B**) near TSS under normal conditions or after transcription inhibition. Samples in the plot are combined replicates after normalization for library size and subtraction of paired input signal. (**D**) Relative distribution for all versus lost AGO2 peaks upon Triptolide treatment across different gene features using the following hierarchy: promoter > exon > intron > intergenic. Promoters are defined as TSS + 1500 bp.

Previous work demonstrated that AGO2 is associated with many active promoters genome-wide. We therefore asked whether ongoing transcription may be required for AGO2 chromatin association by performing AGO2 ChIP-seq analysis after treating Kc167 (Kc) embryonic hemocyte cells with the transcription initiation inhibitor Triptolide^12,13^. We verified transcription inhibition (Figure S1A) and performed ChIP-seq analysis using the 9D6 monoclonal antibody that has been previously validated and used extensively for this purpose by our group and others^1,2,6,14^. In control untreated cells, we observed 3977 AGO2 peaks, and differential binding analysis using the DiffBind algorithm indicates that upon Triptolide treatment, 936 sites are lost while only 13 are gained. Interestingly, we found that 23% of genes upregulated in AGO2 knockdown cells as assayed by neuRNA-seq correspond to an AGO2 peak lost after Triptolide treatment (Fisher’s exact test (FET): odds ratio = 6.5, p < 2.2e-16). To illustrate these findings, we display the *traf4* locus, which corresponds to an AGO2-bound and AGO2 transcription-dependent gene embedded within RED chromatin (Fig. 1A). Further examination of AGO2 signal genome-wide centered at transcription start site (TSS) at lost or unaffected peaks verifies strong loss of AGO2 signal at the TSS specifically after Triptolide treatment (Fig. 1B,C). Furthermore, 73% of lost AGO2 peaks correspond to promoters, indicating strong enrichment compared to other gene features (Binomial test p < 2.2e-16, Fig. 1D). As a control, Western blot analysis showed no changes either for AGO2 or total Pol II (Rpb3 subunit) protein levels after drug treatment (Figure S1B). Together, these results indicate that active transcription is required for AGO2 chromatin association at a significant proportion of promoters.

### AGO2 interacts with nascent RNAs genome-wide

**Figure 2:**
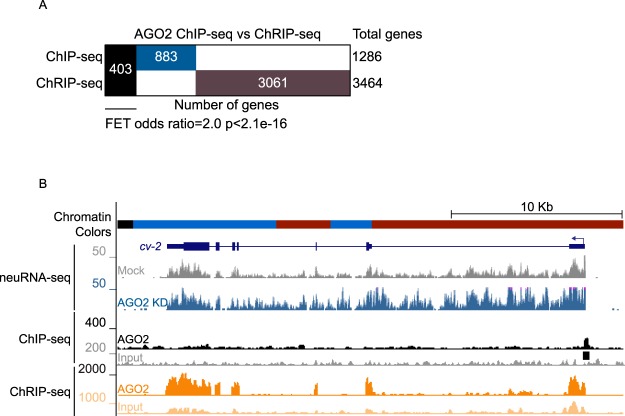
AGO2 associates with nascent RNA genome-wide. (**A**) Binary Heatmap comparing genes positive for AGO2 ChIP-seq or ChRIP-seq. Black bar corresponds to genes bearing AGO2 in both ChIP-seq and ChRIP-seq assays. (**B**) Screenshot showing the example gene *cv-2*, which is up-regulated by neuRNA-seq in AGO2 knockdown and whose TSS is located in active RED chromatin. Black bar corresponds to the AGO2 called ChIP-seq peak.

The dependence on active transcription for AGO2 chromatin association prompted us to evaluate whether AGO2 could be associated with nascent RNA, a hallmark of active transcription. To address this possibility, we performed chromatin RNA immunoprecipitation followed by sequencing (ChRIP-seq) for AGO2 using the same antibody (9D6) used for ChIP-seq analysis. In brief, this assay entails chromatin crosslinking and immunopurification with a final RNA purification, thus allowing the identification of either directly or indirectly associated nascent chromatin-bound RNA^15^. Overall, we found that AGO2 is significantly associated with 3464 chromatin-bound RNAs (Fig. 2). We compared the overlap between ChRIP-seq and ChIP-seq profiles and found that AGO2 ChIP-seq peaks (403 of 1286, 31%) are significantly enriched for genes encoding AGO2-associated ChRIP-seq targets (FET: odds ratio = 2.0, p < 2.2e-16) (Fig. 2A,B). These results support a functional relationship between AGO2 chromatin association and active transcription.

### AGO2 reduces Pol II elongation

**Figure 3:**
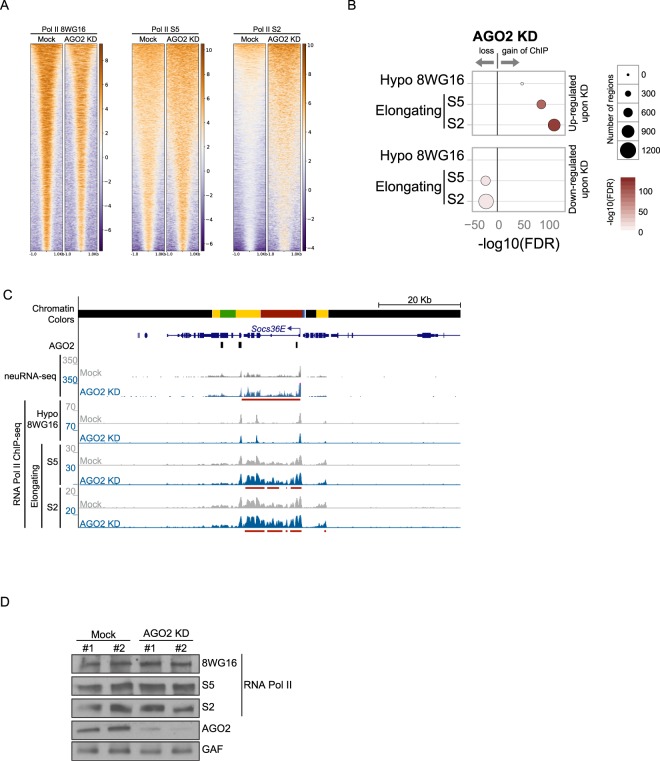
AGO2 reduces Pol II elongation. (**A**) Heatmaps of Pol II 8WG16, Pol II S5 and Pol II S2 peaks in mock-treated cells or upon AGO2 depletion sorted by decreasing average ChIP-seq signal in respective mock-treated cells. The horizontal axis corresponds to distance from peak center for each analyzed factor. (**B**) Differential ChIP-seq analysis for hypophosphorylated (8WG16) and elongating (S5 and S2) Pol II forms on neuRNA-seq affected genes upon depletion of AGO2. Size of circles indicates the number of total genes across the genome that display differential binding (see text for intersection values), and color indicates −log_10_(FDR) where FDR is the p-value from FETs adjusted for multiple comparisons. Only statistically significant results are shown for clarity. (**C**) Screenshot showing the example gene *Socs36E*, which is up-regulated in AGO2 knockdown. AGO2 ChIP peaks (black bars) and ChIP-seq signal of hypophosphorylated, S5, and S2 forms of Pol II are shown. Red bars below signal tracks correspond to statistically significant increases in neuRNA-seq or ChIP-seq signal relative to mock sample. (**D**) Western blot analysis showing no changes in Pol II levels in AGO2 knockdown. AGO2 is efficiently knocked down, and GAF is included as loading control. The number above each lane indicates biological replicate. Representative western blots (cropped) are shown.

Previously, we showed that AGO2 forms a nuclear complex with Pol II, and chromatin association profiles of both factors correlate positively across chromatin states throughout the genome^3^. These findings prompted us to test whether AGO2 influences Pol II occupancy genome-wide. To address this possibility, we first depleted AGO2 by knockdown and then detected differential ChIP-seq peaks compared to control cells for the hypophosphorylated form of the C-terminal domain (CTD) to examine total Pol II (8WG16 antibody, Fig. 3A). We also tested the Serine 5 (S5) and Serine 2 (S2) phosphorylated CTD versions of Pol II to specifically detect elongating forms. Interestingly, only 9 of a total 744 neuRNA-seq up-regulated genes (AGO2-inhibited genes, odds ratio 26) display an increase in hypophosphorylated Pol II (Fig. 3B,C). However, we found a much more dramatic effect on both Pol II S5 and S2 upon depletion of AGO2, where 162 up-regulated genes gain elongating Pol II S5 (odds ratio 11), and 219 genes gain Pol II S2 (odds ratio 11). For the 196 total down-regulated genes, 37 lose elongating Pol II S5 (odds ratio 6.8) and 46 lose Pol II S2 upon depletion of AGO2 (odds ratio 4.8, example shown in Fig. 3C). Importantly, gain of Pol II S5 or S2 is significantly associated with both AGO2 ChRIP-seq peaks and AGO2-attenuated genes (Figures S2 and S3, respectively). No changes were observed for overall Pol II protein levels in AGO2 knockdowns (Fig. 3D).

As a control for the specificity of AGO2 affecting S5 and S2 Pol II forms, we also performed knockdown of LaminB. In contrast to AGO2, LaminB mainly affects the presence of the hypophosphorylated form of Pol II (352 of 1452 neuRNA-seq upregulated genes, odds ratio 6.7) but has a lesser effect on S5 (2 genes, odds ratio 22) or S2 (83 genes, odds ratio 12) phosphorylated versions (Figure S4). Altogether, these results suggest that AGO2 specifically limits the ability of Pol II to elongate.

### AGO2 modulates NELF-E recruitment to affected genes

**Figure 4:**
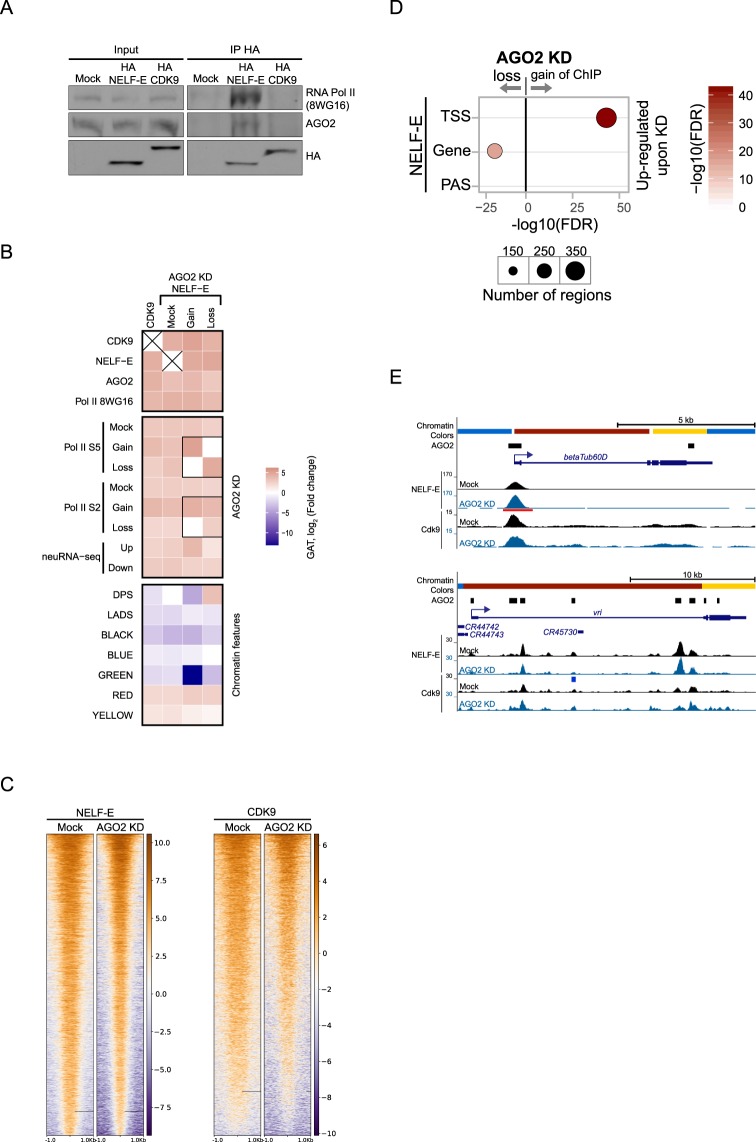
AGO2 modulates NELF-E recruitment to affected genes. (**A**) Western blot showing co-immunoprecipitation of HA-NELF-E, AGO2, and Pol II from Kc nuclear extracts using monoclonal anti-HA antibody. HA-CDK9 does not co-immunoprecipitate AGO2 or hypophosphorylated Pol II. Representative western blots (cropped) are shown. (**B**) Heatmap showing enrichment and depletion of CDK9 and NELF-E ChIP-seq peaks across different factors, chromatin features, AGO2-affected elongating Pol II sites, and AGO2-affected neuRNA-seq genes. In addition, NELF-E differentially bound peaks upon AGO2 KD are included for comparison. Colormap represents the log_2_ fold change as reported by the Genomic Association Test (GAT), where negative (blue) indicates depletion and positive (red) indicates enrichment. Self-self comparisons are indicated by an X. (**C**) Heatmaps of NELF-E and CDK9 peaks in mock-treated cells or upon AGO2 depletion sorted by decreasing average ChIP-seq signal in respective mock-treated cells. The horizontal axis corresponds to distance from peak center for each analyzed factor. (**D**) Differential ChIP-seq analysis for NELF-E on neuRNA-seq up-regulated genes upon depletion of AGO2. Gene body is defined as any gene region that does not overlap with TSS or PAS (polyadenylation site). Size of circles indicates the number of total genes across the genome that display differential binding (see text for intersection values), and color indicates −log_10_(FDR) where FDR is the p-value from FETs adjusted for multiple comparisons. Only statistically significant results are shown for clarity. (**E**) Screenshot showing the example genes *betaTub60D* and *vri*, which are up-regulated in AGO2 knockdown. Their corresponding TSSes are located within RED chromatin. AGO2 ChIP peaks (black bars) and ChIP-seq signals of NELF-E and CDK9 are shown. Red and blue bars below signal tracks correspond to differential binding and statistically significant increases and decreases, respectively, in ChIP-seq signal relative to mock sample.

The above results led us to investigate whether AGO2 could modulate chromatin association of factors that control the transcription cycle, such as NELF-E and CDK9. In brief, NELF-E has been implicated in transcriptional pausing whereas CDK9 phosphorylates Pol II at S2, thus contributing to transcriptional elongation^16^. First, we transfected NELF-E and CDK9 HA-tagged constructs into Kc cells and performed immunoprecipitation from nuclear extracts followed by Western blotting against AGO2. In concordance with our previous AGO2 mass spec analysis of nuclear interacting factors^3^, we found that NELF-E but not CDK9 associates with AGO2 (Fig. 4A).

Next, we performed ChIP-seq analysis of both factors in Kc cells using antibodies against the endogenous proteins. These antibodies have been previously validated for ChIP assays^9,10^, and we obtained 9252 total peaks for NELF-E and 8416 peaks for CDK9. As expected, cross-comparisons with different factors and chromatin features showed enrichment for Pol II and active chromatin (RED, YELLOW) genome-wide (Fig. 4B). We found that both factors also significantly overlap with AGO2 ChIP-seq genome-wide (GAT: 62% of NELF-E occupancy, odds ratio 11 p <= 1.0e-3, 74% of CDK9 occupancy, odds ratio 19 p <= 1.0e-3). Importantly, we observed that occupancy of either protein is also highly significantly associated with both AGO2-inhibited genes (FET: 565 of 744 genes for NELF-E, odds ratio 5.3 p < 2.2e-16, 374 genes for CDK9, odds ratio 4.8 p < 2.2e-16) as well as AGO2-promoted genes (FET: 180 of 196 genes for NELF-E, odds ratio 18 p < 2.2e-16, 94 genes for CDK9, odds ratio 4.1 p < 2.2e-16). These results are consistent with the hypothesis that AGO2-dependent genes are regulated on the level of transcriptional elongation.

To evaluate whether AGO2 might control chromatin binding for either protein, we performed differential ChIP-seq analysis of NELF-E and CDK9 upon depletion of AGO2. Interestingly, we found that depletion of AGO2 results in increased NELF-E binding at 249 genes and decreased binding at 138 genes across the genome (Fig. 4C). In addition, we found that gain/loss of NELF-E upon depletion of AGO2 is highly correlated with differential binding for Pol II S5 and, to a lesser extent, with Pol II S2 (Fig. 4B). Along this line, we observed that AGO2 up-regulated genes are significantly enriched for a gain of NELF-E binding at TSSes (FET: NELF-E gain: 76 genes, odds ratio 11, Figs 4C,D, S3). However, less obvious loss of NELF-E was also detected on gene bodies of upregulated genes (FET: 35 genes, odds ratio 8.0, Fig. 4D). Furthermore, gain of NELF is also significantly associated with AGO2 ChRIP peaks genome-wide (Figure S2). On the other hand, CDK9 showed no change in chromatin association, indicating specificity of the effect on NELF-E. We also attempted to evaluate CDK7, the kinase that phosphorylates Pol II on S5^17^, but we were unable to obtain a chromatin association profile by ChIP-seq. Together, these results suggest that AGO2 modulates NELF-E but not CDK9 chromatin recruitment.

## Discussion

In this study, we performed a variety of genome-wide assays to study requirements for AGO2 chromatin association and the function of AGO2 in transcription regulation. ChIP-seq analysis upon transcription inhibition showed that AGO2 chromatin association at promoters depends on active transcription. Strikingly, AGO2 attenuates active transcription by preventing Pol II elongation. Finally, AGO2 also affects the chromatin association of NELF-E but not the elongation factor CDK9. Altogether, these results provide important insights into AGO2 chromatin association and its mechanistic role in controlling active transcription by modulating the transcription cycle.

### AGO2 is recruited to promoters in a transcription-dependent manner

**Figure 5:**
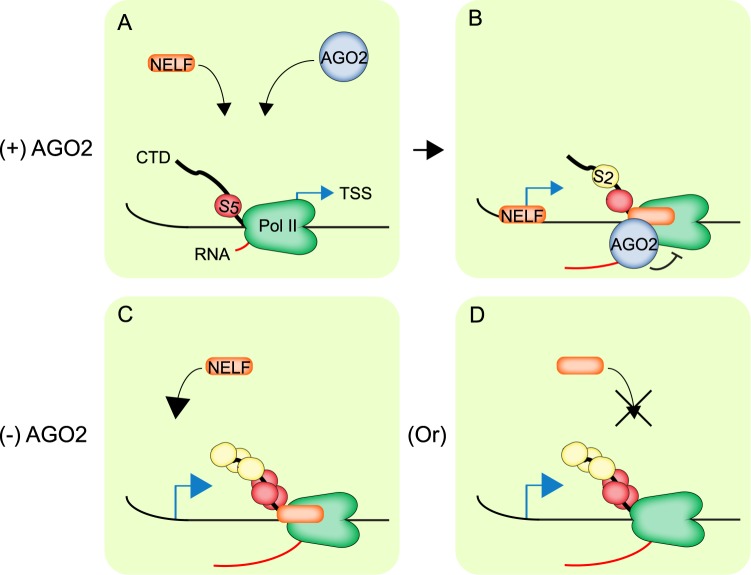
A model for AGO2 recruitment and attenuation of active transcription. (**A**) Under normal conditions, AGO2 is recruited to active promoters likely by interacting with the transcription initiation machinery. (**B**) Once polymerase transitions into elongation, AGO2 may be transferred to mRNA and attenuate transcription. Alternatively, AGO2 association with nascent RNAs could be mediated by Pol II and/or other factors. (**C**,**D**) In the absence of AGO2, there is an increase of transcription elongation and CTD phosphorylation of Pol II on S5 and S2, in addition to either (**C**) gain of NELF at the TSS or (**D**) loss of NELF over the gene body.

A recurrent question in the field is to understand the mechanism driving AGO2 association into chromatin given the absence of a DNA-binding motif. In this regard, we previously showed that AGO2 chromatin-association correlates positively with Pol II binding genome-wide^3^. This result raises the possibility that AGO2 might be recruited to promoters as a result of interaction with the transcription machinery. Accordingly, we found that AGO2 chromatin binding is impaired upon Triptolide treatment, a drug that inhibits transcription initiation and reduces Pol II recruitment^12,13,18^. In addition, ChRIP-seq experiments showed that AGO2 associates with the nascent RNA of a large number of genes to which it is recruited at the chromatin level. Additional nascent mRNAs with which *Drosophila* AGO2 associates might correspond to alternative splicing events^2^. In support of this hypothesis, it has been recently shown in human cancer cells that AGO2 binds directly to nascent tRNAs and associates with chromatin from which those tRNAs are transcribed^19^. Similarly, human AGO2 specifically binds the sense strand of nascent tRNAs in a small RNA-and DICER-independent manner^19^. In addition, it has been shown that human AGO2 contains its own mRNA binding region, and *in silico* analysis suggests that this feature is evolutionarily conserved^20^. However, we cannot discard the possibility that AGO2 interacts indirectly with nascent RNA by interacting with Pol II and/or NELF at promoters. Overall, we propose that the transcription machinery helps to recruit AGO2 to chromatin and that in some cases AGO2 can subsequently be transferred to nascent mRNAs (Fig. 5).

### AGO2 modulates active transcription by controlling Pol II elongation

We showed that AGO2 plays a repressive role in transcription genome-wide by limiting Pol II elongation. In this regard, AGO2-depleted cells mainly showed differences in levels of S5 and S2 phosphorylated elongating forms. In contrast, LaminB depletion resulted in substantial increases in recruitment of initiating hypophosphorylated Pol II, consistent with increased accessibility to the transcription machinery due to effects on chromatin topology. Our results could be interpreted as AGO2 acting downstream of transcription initiation and constraining Pol II elongation (Fig. 5). Nevertheless, in AGO2-depleted cells, corresponding changes in elongating Pol II are observed for both up-regulated and down-regulated genes. These results suggest that AGO2 can exert either positive or negative effects on transcription elongation depending on the chromatin context, as has previously been described for NELF^11^. We found that AGO2 and NELF can associate physically, either directly or indirectly, and their genome-wide localization patterns overlap substantially. Although both factors predominantly repress transcription genome-wide, AGO2 and NELF are preferentially associated with actively transcribed genes, suggesting a role in attenuating but not fully repressing transcription. Depletion of AGO2 promotes numerous instances of either gain of NELF at the TSS but also loss of NELF over gene bodies, and these changes in NELF occupancy frequently but do not always correlate with a detectable change in nascent transcription. Overall, these findings raise the possibility that AGO2 might modulate the recruitment and/or activity of NELF to fine-tune transcription at active promoters.

In contrast, AGO2 does not affect the recruitment of CDK9, likely due to lack of association between both factors. However, the relatively low percentage of AGO2-dependent genes that gain/lose NELF-E (10%/7%, respectively) suggest that AGO2 might exert its role on the transcription cycle by affecting other elongation factors, for instance, CTD phosphatases. Alternatively, AGO2 might modulate Pol II phosphorylation by affecting a pre-mRNA processing step coupled to CTD phosphorylation^21^. Further analysis will be required to shed light on the factors/processes modulated by AGO2 that in turn affect the phosphorylation status of Pol II and its transition into elongation.

## Conclusion

It is worth consideration that the dependence of AGO2 on transcription for its chromatin association as well as its role in controlling Pol II recruitment are evolutionarily conserved. It has been shown that AGO1 (orthologue of AGO2 in mammals) is associated with active promoters and enhancers in humans genome-wide^22,23^. Moreover, AGO1 chromatin association at active enhancers is impaired upon transcription inhibition^22^. In addition, AGO1 is significantly associated with oncogene promoters in cancer cells and affects Pol II recruitment^23^. As a consequence, AGO1 depletion reduces proliferation of tumorigenic cell lines, indicating that AGO1 may serve as a useful therapeutic target in treatment of cancers. In summary, our results contribute to a better mechanistic understanding of the role of AGO2 in transcription regulation and may inform future avenues of research with respect to AGO1 and control of cancer progression.

## Materials and Methods

### Cell lines

Kc167 cells were grown in CCM3 media (Thermo Scientific HyClone). Cells were maintained at 25 °C. For transcription inhibition assays, cells were treated with 10 μM Triptolide for 1 h.

### Knockdowns using dsRNA and siRNA

Amplicons used for dsRNA knockdowns were designed based on recommendations from the *Drosophila* RNAi Screening Center. Templates were PCR-amplified from genomic DNA using primers containing the T7 promoter sequence. *In vitro* transcription of PCR templates using the MEGAscript T7 kit (Ambion) was used to produce dsRNAs, and these were purified by phenol-chloroform extraction. Transfections using 2 μg of dsRNA or 100 pmol of siRNA, or no dsRNA/siRNA as mock treatment, were performed using Cell Line Nucleofector kit V (Amaxa Biosystems) transfection reagent using the G-30 program. Three days after transfection, cells were collected and knockdown efficiency was confirmed by Western blotting.

### NELF-E and CDK9 constructs

To generate NELF-E and CDK9 constructs, NELF-E and CDK9 cDNAs were cloned into p-ENTR/D-TOPO gateway vector (Invitrogen). These constructs were recombined into the pAFHW (Carnegie vectors) expression vector to generate N-terminal 3xFlag, 3XHA fusion constructs.

### Co-immunoprecipitation

Approximately 5 × 10^7^ cells were harvested and washed twice with cold PBS. Cells were lysed for 5 min on ice in lysis buffer (Tris-HCl pH 7.4 30 mM, 100 mM NaCl, 10 mM MgCl_2_, 0.1% Triton X-100) supplemented with Complete EDTA free protease inhibitors (Roche) and centrifuged for 5 min at 500 × *g* at 4 °C. The supernatant was removed and pellets were washed once with lysis buffer, resuspended in IP buffer (50 mM Tris-HCl pH 7.4, 150 mM NaCl, 0.3 mM MgCl_2_, 0.3% Triton X-100), incubated 10 min on ice and sonicated 10 cycles for 30 sec, to shear DNA (nuclear fraction). Nuclear extracts were incubated with a polyclonal antibody against HA (H6908) from Sigma, and allowed to bind for 1 h at 4 °C. Next, 50 μL of prewashed Protein G bead 50% slurry was added. After incubation for 2 h, unbound supernatant was removed, and the beads were washed once in IP buffer and twice in TBS (50 mM Tris-HCl pH 7.4, 150 mM NaCl). The bound protein was eluted in sample buffer by boiling, separated by using SDS-PAGE, transferred to nitrocellulose in 10 mM CAPS, pH 11, and detected by Western blotting. Proteins were detected with the SuperSignal substrate (Pierce).

### ChIP

Approximately 1–3 × 10^7^ cells were fixed by addition of 1% formaldehyde to cell media for 10 min at RT with gentle agitation. Formaldehyde was quenched by addition of glycine to 0.125 M with gentle agitation for 5 min at RT. Cells were pelleted at 2000 × *g*, washed twice in PBS, and resuspended in 0.8 mL ice–cold cell lysis buffer (5 mM PIPES pH 8, 85 mM KCl, 0.5% NP-40) supplemented with Complete protease inhibitors (Roche), incubated on ice 10 min, and pelleted by centrifugation at 2000 × *g* for 5 min at 4 °C. Next, the supernatant was removed and pellets were resuspended in 1 mL nuclear lysis buffer (50 mM Tris-HCl pH 8, 10 mM EDTA.Na2, 1% SDS) and incubated for 10 min at 4 °C. Afterwards, 0.5 mL of IP dilution buffer was added (16.7 mM Tris-HCl pH 8, 1.2 mM EDTA, 167 mM NaCl, 1.1% Triton X-100, 0.01% SDS), and chromatin was fragmented to an average size of 300 bp by using PicoBioruptor (Diagenode) using 10 cycles of 30 s on plus 30 s off, maximum output. Samples were centrifuged at max speed for 10 min at 4 °C, and the supernatant (sheared chromatin) was saved at −80 °C. Chromatin was diluted to 1:5 with IP buffer, and the assayed antibody in addition to 50 μL of prewashed protein A/G 50% slurry was added and rotated overnight at 4 °C. The next day, beads were washed as follows:

- 3X Low salt IP dilution buffer (20 mM Tris-HCl pH 8, 2 mM EDTA.Na2, 150 mM NaCl, 1% Triton X-100, 0.1% SDS).
- 3X High salt IP dilution buffer (20 mM Tris-HCl pH 8, 2 mM EDTA.Na2, 500 mM NaCl, 1% Triton X-100, 0.1% SDS).
- 2X times LiCl buffer (10 mM Tris-HCl pH 8, 1 mM EDTA.Na2, 250 mM LiCl, 1% NP-40, 1% DOC).

Chromatin was eluted twice with 200 μL of elution buffer (100 mM NaHCO_3_, 1.0% SDS) for 30 min at 65 °C each and further incubated overnight at 65 °C with 38 μL of decrosslinking solution (2.6 M NaCl, 0.1 M EDTA, 260 mM Tris-HCl pH 8). After de-crosslinking, samples were treated with Proteinase K for 2 h at 50 °C and then combined with 1 vol of phenol/chloroform/isoamyl alcohol (25:24:1), vortexed 15 s, and centrifuged for 5 min at 10,000 × *g*. The top layer was transferred to a new tube, and the procedure was repeated using 1 vol chloroform. The top layer was collected and subsequently precipitated with 0.1 vol of 3M NaOAc pH 5.2 and 2.5 vol of 100% ethanol supplemented with 2 μL of Glycoblue (Ambion). After incubating 30 min at −80 °C, samples were centrifuged 20 min at 4 °C at 10,000 × *g*. Pellets were washed with 70% ethanol and centrifuged 5 min at 4 °C at 10,000 × *g*. Pellets were air dried at RT prior to resuspension in 10 μL of dH2O. Samples for ChIP-seq were prepared according to the manufacturer’s protocol with TruSeq adapters (Illumina). All samples were sequenced with HiSeq2500 (Illumina) at the NIDDK Genomics Core Facility by 50 bp single-end sequencing.

### ChRIP-seq

ChRIP-seq protocol was based on the ChIP workflow with minor modifications. Lysis buffer included RNasin (Promega) in addition to protease inhibitors. Beads were pre-blocked with 0.5% of tRNA and 0.2% BSA for 1 h. After the last LiCl wash, beads were resuspended in 100 μL of 50 mM Tris-Cl pH 7.0, 5 mM EDTA and 1% SDS and de-crosslinked at 65 °C for 2 h. Next, beads were treated with DNAse I for 15 min at 37 °C, and total RNA was extracted with TRIzol (Invitrogen). RNA-seq libraries were prepared with Ovation RNA-seq Systems 1–16 for Model Organisms (Nugen). Differential enrichment for IP over input was determined as previously described using DESeq2^3^. All samples were sequenced with HiSeq2500 (Illumina) at the NIDDK Genomics Core Facility by 50 bp single-end sequencing.

### qRT–PCR

Total RNA was isolated from cells using TRizol reagent (Invitrogen) following the manufacturer’s protocol. Reverse transcription of 0.5–1 μg of total RNA was performed using random hexamers as primers and SuperScript III reverse transcriptase (Invitrogen) using the manufacturer’s protocol. Transcript levels were quantified in the linear amplification range by real-time PCR using HotStart-IT SYBR green qPCR Master Mix (USB Corporation) by calibration to a standard curve of genomic DNA to account for differences in primer efficiencies.

### ChIP-seq

Trimming reads for adapters and light quality trimming was performed with cutadapt v1.15 using the parameters “–quality-cutoff 20 -a AGATCGGAAGAGC–minimum-length = 25”. Reads were aligned to the dm6 reference genome using bowtie2 v2.3.3.1^24^ with default parameters. Multimapping reads were removed using the “view” program of samtools v1.7^25^ with parameter “-q 20”. The Picard tools v2.17.0 (http://broadinstitute.github.io/picard/) program MarkDuplicates was used to remove PCR duplicates from mapped reads.

Punctate peaks for AGO2, NELF-E, and CDK9, and broad regions for Pol II were called with the MACS2 v2.1.1.20160309 callpeak program^26^ and https://github.com/taoliu/MACS). Each replicate was called separately, and then an additional pooled peak call set was generated by providing all input and IP samples from both replicates to MACS2 simultaneously. For both AGO2 and Pol II we used the non-default parameters “–gsize dm–bdg–SPMR”. For Pol II we additionally included the parameter “–broad”. The signal bedGraph outputs from the pooled peak-calling runs were used in screenshots for figures. We selected final peaks as those peaks in the pooled peak-calling run that were also found in at least one of the individual replicates. Specifically, using BEDTools v2.25.0 and pybedtools v0.7.9^27,28^ we used “pooled.intersect([replicates], u = True).sort().merge()” where “pooled” points to the pooled peak calls and “[replicates]” is the list of individual peak calls for each replicate.

### Differential ChIP-seq

For detecting differential ChIP-seq binding, we used the Diffbind v1.16.3 Bioconductor/R package^29^ using the config object “data.frame(RunParallel = TRUE,DataType = DBA_DATA_FRAME, AnalysisMethod = DBA_EDGER, bCorPlot = FALSE, bUsePval = FALSE, fragmentSize = 300)” and otherwise used defaults. Input files consisted of the final peak calls described above, and the IP and input BAM files for each replicate as described above with multimappers and duplicates removed. The final results were exported with the dba.report function with parameters “th = 1, bCalled = TRUE, bNormalized = TRUE, bCounts = TRUE” and final differentially gained or lost peaks were those that had a log2 fold change of >0 or <0 respectively and an FDR < 0.05.

### External data

Chromatin colors from^8^ were downloaded from GEO accession GSE22069 and lifted over to the dm6 assembly using liftOver from the UCSC tools^30^.

### Fisher’s exact tests

All FETs used the implementation in the Python scipy package (scipy.stats.fisher_exact). When comparing intervals (peaks, LADs) with genes, we considered the entire gene body. Reported p-values are two-tailed. When multiple tests were performed, we applied the Benjamini-Hochberg multiple test correction (FDR) as implemented in the statsmodels Python package (statsmodels.stats.multitest.fdrcorrection). Where FDR is displayed in heatmaps, if the odds ratio was <1 then the sign of the corresponding FDR was flipped such that negative values indicate depletion and positive values indicate enrichment. For FETs comparing differentially expressed genes with differential ChIP peaks, comparisons were performed between up or downregulated genes versus unchanged genes. Odds ratios and FDRs are reported in text or corresponding figure.

### Colocalization

For computing colocalization between sets of intervals, we used the Genomic Association Test v1.2.2 framework^31^ using the dm6 assembly as the domain or workspace and otherwise default parameters. In the heatmaps showing log2 fold change enrichment, any comparisons where the q-value was <0.05 was reset to have a log2 fold change of zero, and any self-self comparisons were set to zero.

### Deposited Data

https://www.ncbi.nlm.nih.gov/geo/query/acc.cgi?acc=GSE116889.

## Electronic supplementary material

Supplementary information
