# Supplementary material for: Argonaute2 attenuates active transcription by limiting RNA Polymerase II elongation in Drosophila melanogaster
Source: Sci Rep. 2018 Oct 24;8:15685. doi: 10.1038/s41598-018-34115-1 (PMC6200757; doi:10.1038/s41598-018-34115-1)
Supplement: Supplementary file 1 — Supplementary information [file 41598_2018_34115_MOESM1_ESM.pdf]

Supplementary Information for:

Argonaute2 attenuates active transcription by limiting RNA Polymerase II elongation in *Drosophila melanogaster*

Ezequiel Nazer[1,2], Ryan K. Dale[2], Cameron Palmer[2], and Elissa P. Lei[1,2*]

[1]Nuclear Organization and Gene Expression Section

[2]Laboratory of Cellular and Developmental Biology, National Institute of Diabetes and Digestive and Kidney Diseases, National Institutes of Health, 9000 Rockville Pike, Bethesda, MD 20892, USA

*Correspondence: leielissa@niddk.nih.gov

Supplemental Figure Legends

Figure S1 (related to figure 1)

A. Validation by qRT-PCR for Triptolide transcription inhibition in Kc167 cells. Levels of *betaTub60D* pre-mRNA in triptolide-treated cells are relative to mock-treated cells and normalized to *U6* snRNA. Error bars correspond to 95% confidence interval of four experiments.

B. Western blot analysis showing no changes either for total Pol II or AGO2 levels upon Triptolide treatment. Representative western blots (cropped) are shown.

Figure S2 (related to figures 3, 4)

Scatterplot comparing gain of Pol II 8WG16, S5, S2 or NELF-E peaks upon AGO2 knockdown and AGO2 ChRIP-seq peaks (red). Genes gaining the indicated factor at AGO2 ChRIP peaks are colored in orange. FET *p*-values and odds ratios indicate significance of the overlap between gain of the indicated factor peaks and AGO2 ChRIP-seq peaks.

Figure S3 (related to figures 3, 4)

Scatterplot comparing gain of Pol II 8WG16, S5, S2 or NELF-E peaks upon AGO2 knockdown and neuRNA-seq AGO2-dependent genes (up=red, down=blue). Genes gaining the indicated factor at AGO2-dependent genes are colored in orange. FET *p*-values and odds ratios indicate significance of the overlap between gain of the indicated factor peaks and AGO2-inhibited genes.

Figure S4 (related to figure 3)

A. Differential ChIP-seq analysis for hypophosphorylated (8WG16) and elongating (S5 and S2) Pol II forms on neuRNA-seq affected genes upon depletion of LaminB. Size of circles indicates the number of total genes across the genome that display differential binding (see text for intersection values), and color indicates $-\log_{10}$(FDR) where FDR is the p-value from FETs adjusted for multiple comparisons. Only statistically significant results are shown for clarity.

B. Screenshot showing the example gene *Socs36E*, which is up-regulated in LaminB knockdown. AGO2 ChIP peaks (black bars) and ChIP-seq signal of hypophosphorylated, S5, and S2 forms of Pol II are shown. Red bars below signal tracks correspond to statistically significant increases in neuRNA-seq or ChIP-seq signal relative to mock sample.

Supplemental table legends

Table S1 (related to Materials and Methods). Antibodies used in this study

Table S2 (related to Materials and Methods). Oligos used in this study

A

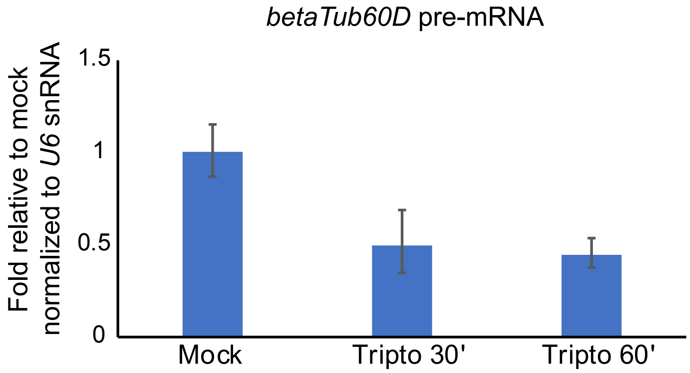
betaTub60D pre-mRNA
Fold relative to mock normalized to *U6* snRNA
1.5
1
0.5
0
Mock
Tripto 30'
Tripto 60'

B

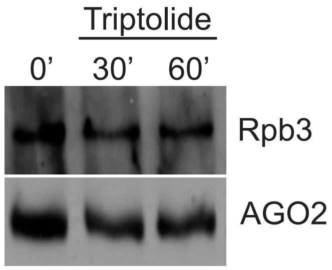
Triptolide
0’
30’
60’
Rpb3
AGO2

Figure S2

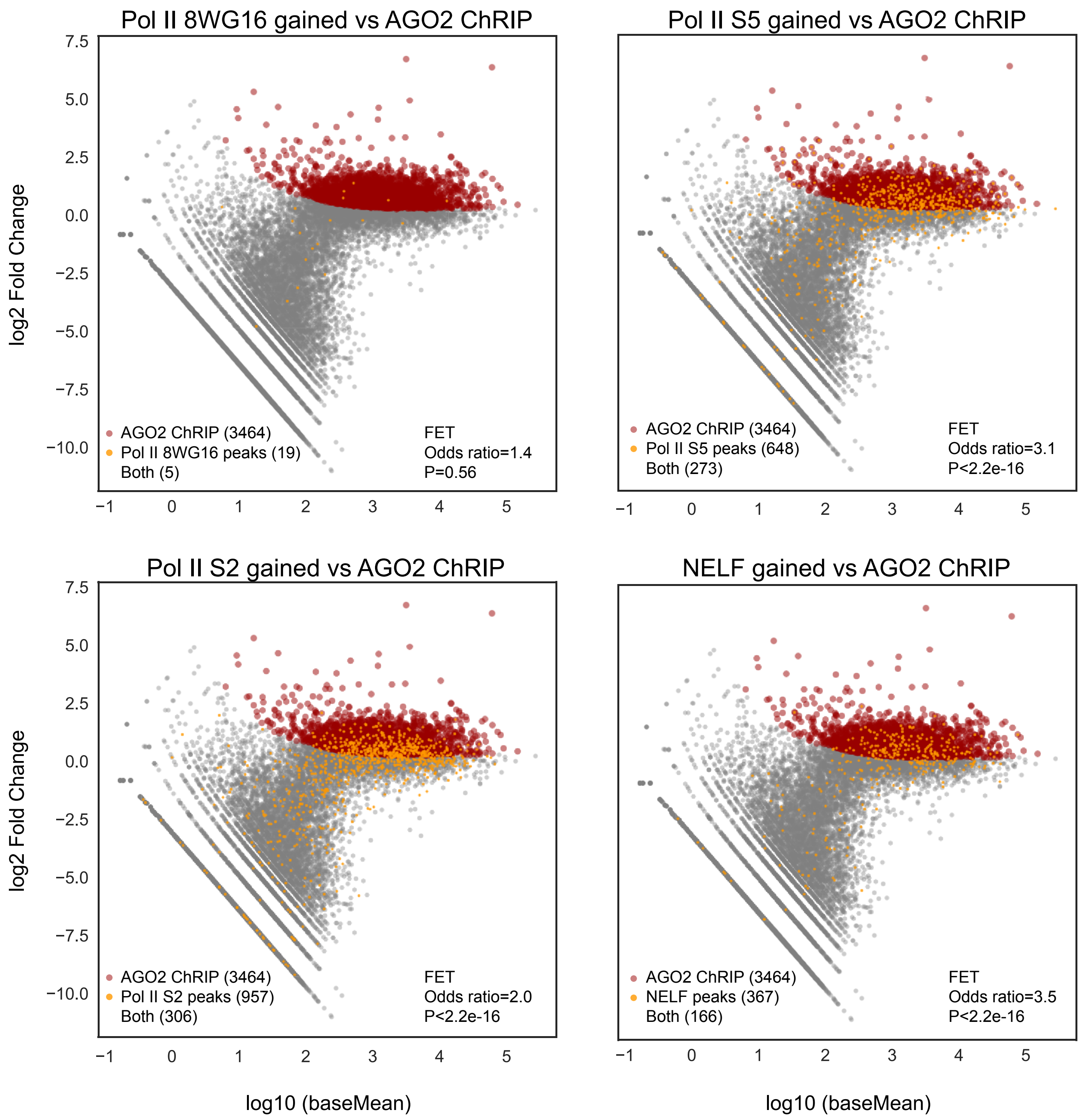

Pol II 8WG16 gained vs AGO2 ChRIP
AGO2 ChRIP (3464)
Pol II 8WG16 peaks (19)
Both (5)
FET
Odds ratio=1.4
P=0.56
Pol II S5 gained vs AGO2 ChRIP
AGO2 ChRIP (3464)
Pol II S5 peaks (648)
Both (273)
FET
Odds ratio=3.1
P<2.2e-16
Pol II S2 gained vs AGO2 ChRIP
AGO2 ChRIP (3464)
Pol II S2 peaks (957)
Both (306)
FET
Odds ratio=2.0
P<2.2e-16
NELF gained vs AGO2 ChRIP
AGO2 ChRIP (3464)
NELF peaks (367)
Both (166)
FET
Odds ratio=3.5
P<2.2e-16
log2 Fold Change
log10 (baseMean)
7.5
5.0
2.5
0.0
−2.5
−5.0
−7.5
−10.0
−1
0
1
2
3
4
5

Figure S3

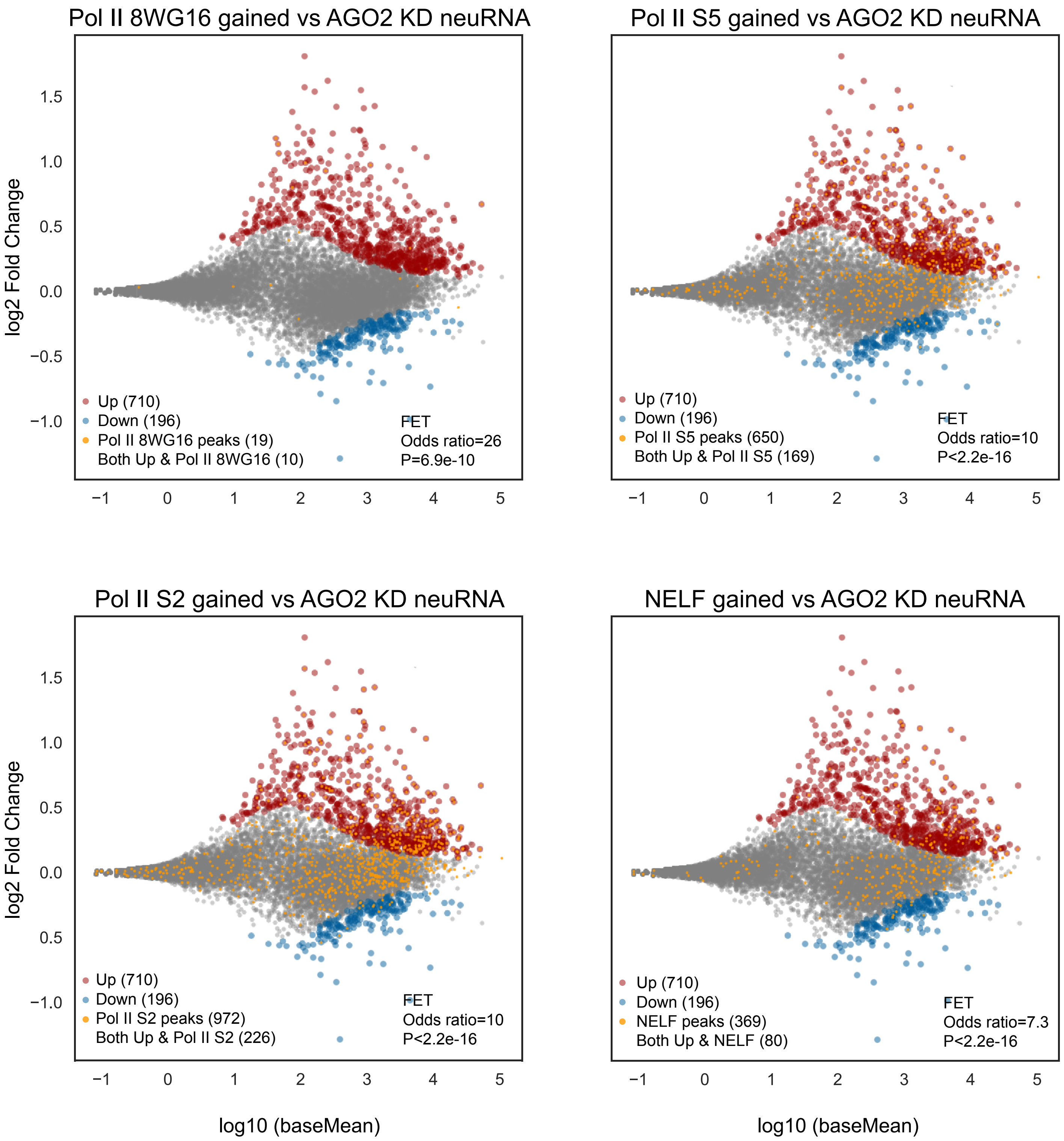

Pol II 8WG16 gained vs AGO2 KD neuRNA
log2 Fold Change
1.5
1.0
0.5
0.0
−0.5
−1.0
−1
0
1
2
3
4
5
Up (710)
Down (196)
Pol II 8WG16 peaks (19)
Both Up & Pol II 8WG16 (10)
FET
Odds ratio=26
P=6.9e-10
Pol II S5 gained vs AGO2 KD neuRNA
Up (710)
Down (196)
Pol II S5 peaks (650)
Both Up & Pol II S5 (169)
FET
Odds ratio=10
P<2.2e-16
Pol II S2 gained vs AGO2 KD neuRNA
log2 Fold Change
1.5
1.0
0.5
0.0
0.5
−1.0
Up (710)
Down (196)
Pol II S2 peaks (972)
Both Up & Pol II S2 (226)
FET
Odds ratio=10
P<2.2e-16
NELF gained vs AGO2 KD neuRNA
Up (710)
Down (196)
NELF peaks (369)
Both Up & NELF (80)
FET
Odds ratio=7.3
P<2.2e-16
log10 (baseMean)
log10 (baseMean)

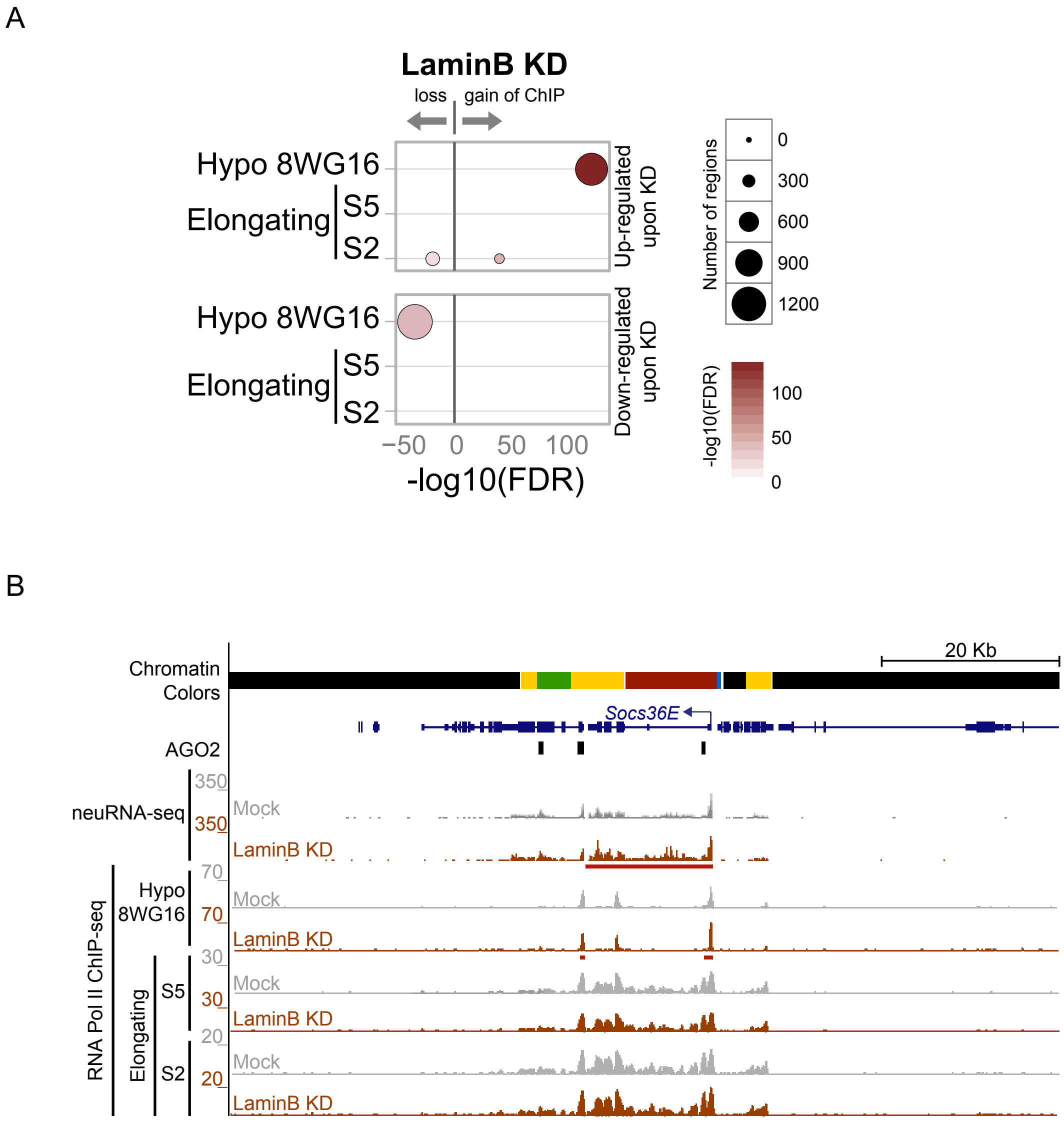
A
LaminB KD
loss
gain of ChIP
Hypo 8WG16
Elongating
S5
S2
Up-regulated upon KD
Down-regulated upon KD
−50
0
50
100
-log10(FDR)
Number of regions
0
300
600
900
1200
-log10(FDR)
100
50
0
B
Chromatin Colors
20 Kb
Socs36E
AGO2
neuRNA-seq
350
Mock
LaminB KD
RNA Pol II ChIP-seq
Hypo 8WG16
70
30
20
Elongating
S5
S2

Table S1 (related to Materials and Methods). Antibodies used in this study

Name	Application	Lab/company	Catalog
AGO2 9D6	ChIP-seq	Siomi	N/A
AGO2 Mueller	ChIP-seq	Mueller	N/A
AGO2 Liu	Wb	Liu	N/A
CDK9	ChIP-seq	Nakamura	N/A
NELF-E	ChIP-seq	Gilmour	N/A
RNA Pol II Rpb3	Wb	Adelman	N/A
RNA Pol II 8WG16	ChIP-seq; Wb	Biolegend	920102
RNA Pol II S5	ChIP-seq; Wb	Abcam	Ab5408
RNA Pol II S2	ChIP-seq; Wb	Abcam	Ab5095
HA	IP	Sigma	H6908
HA	Wb	Santa Cruz	sc57592
GAF	Wb	Cavalli	N/A

Wb: western blot
IP: immunoprecipitation

Table S2 (related to Materials and Methods). Oligos used in this study

Primer	Gene	Application	Sequence
siAGO2	CG7439	siRNA knockdowns	CAACCACAGCAGCUGCAACdTdT
siAGO2	CG7439	siRNA knockdowns	GUUGCAGCUGCUGUGGUUGdTdT
T7LaminB For	CG6944	dsRNA knockdowns	TAATACGACTCACTATAGGGAGAGGAGAACACCATTCAGAGTC
T7LaminB For	CG6944	dsRNA knockdowns	TAATACGACTCACTATAGGGAGACCATAAGGTCCTGGTACTCC
Fv_NELF_E_pentry	CG5994	Cloning in P-entry TOPO	caccATGGTTTACATACACTTCCCCAA
Rv_NELF_E_pentry	CG5994	Cloning in P-entry TOPO	CTAGAGCAAGAAGTCTTCATCGT
Fv_CDK9_pentry	CG5179	Cloning in P-entry TOPO	caccATGGCGCACATGTCCC
Rv_CDK9_pentry	CG5179	Cloning in P-entry TOPO	CTACCAAACCCGGTCAATCA
Fv_betatub60D_premRNA	CG3401	qRT-PCR	AAATCGGCGCTAAGgtaggt
Rv_betatub60D_premRNA	CG3401	qRT-PCR	cagccgatcattttggaaac
Fv_U6	CR31379	qRT-PCR	GCTTCGGCAGAACATATACT
Rv_U6	CR31379	qRT-PCR	AAAAATGTGGAACGCTTCAC
